# Supplementary material for: A whole-animal phenotypic drug screen identifies suppressors of atherogenic lipoproteins
Source: eLife. 2026 Aug 27;14:RP105314. doi: 10.7554/eLife.105314 (PMC13521566; doi:10.7554/eLife.105314)
Supplement: Supplementary file 2 [file elife-105314-code1.zip › JHDL_primary_result/CYTOCHALASIN N/CYTOCHALASIN N_JHDL_5_017_017_2019_05_17_11_22.pdf]

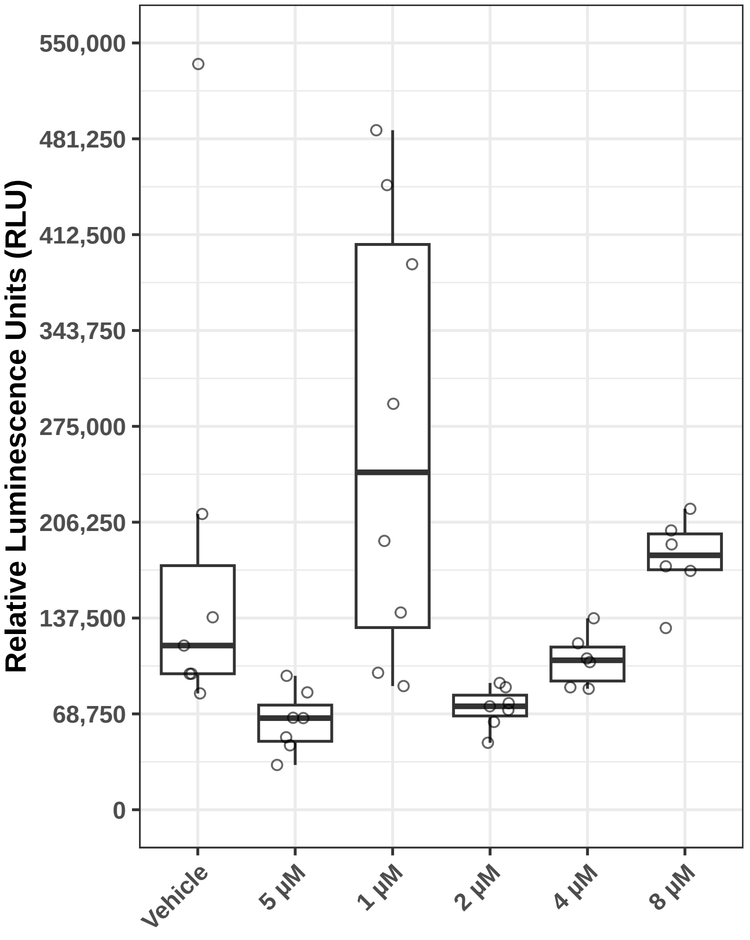
Relative Luminescence Units (RLU)
550,000
481,250
412,500
343,750
275,000
206,250
137,500
68,750
0
Vehicle
5 µM
1 µM
2 µM
4 µM
8 µM
